# The alteration of RhoA geranylgeranylation and Ras farnesylation breaks the integrity of the blood–testis barrier and results in hypospermatogenesis

**DOI:** 10.1038/s41419-019-1688-9

**Published:** 2019-06-06

**Authors:** Ruilou Zhu, Jiangnan Wang, Tianxiang Feng, Xuechun Hu, Chen Jiang, Xiuxing Wang, Kang Li, Yongjuan Sang, Yue Hua, Haixiang Sun, Bing Yao, Chaojun Li

**Affiliations:** 1 MOE Key Laboratory of Model Animals for Disease Study, Model Animal Research Center and Jiangsu Key Laboratory of Molecular Medicine cell biology, the Medical School of Nanjing University, Nanjing, China; 2 Collaborative Innovation Platform for Reproductive Biology and Technology of the Medical School of Nanjing University, Nanjing, China; 3 Center of Reproductive Medicine, Nanjing Jinling Hospital, the Medical School of Nanjing University, Nanjing, China; 4 Reproductive Medicine Center, The Affiliated Drum Tower Hospital, the Medical School of Nanjing University, Nanjing, China

**Keywords:** Disease model, Infertility

## Abstract

Non-obstructive azoospermia (NOA) severely affects male infertility, however, the deep mechanisms of this disease are rarely interpreted. In this study, we find that undifferentiated spermatogonial stem cells (SSCs) still exist in the basal compartment of the seminiferous tubules and the blood–testis barrier (BTB) formed by the interaction of neighbor Sertoli cells (SCs) is incomplete in NOA patients with spermatogenic maturation arrest. The adhesions between SCs and germ cells (GCs) are also broken in NOA patients. Meanwhile, the expression level of geranylgeranyl diphosphate synthase (*Ggpps*), a key enzyme in mevalonate metabolic pathway, is lower in NOA patients than that in obstructive azoospermia (OA) patients. After *Ggpps* deletion specifically in SCs, the mice are infertile and the phenotype of the SC-*Ggpps*^−/−^ mice is similar to the NOA patients, where the BTB and the SC–GC adhesions are severely destroyed. Although SSCs are still found in the basal compartment of the seminiferous tubules, fewer mature spermatocyte and spermatid are found in SC-*Ggpps*^−/−^ mice. Further examination suggests that the defect is mediated by the aberrant protein isoprenylation of RhoA and Ras family after *Ggpps* deletion. The exciting finding is that when the knockout mice are injected with berberine, the abnormal cell adhesions are ameliorated and spermatogenesis is partially restored. Our data suggest that the reconstruction of disrupted BTB is an effective treatment strategy for NOA patients with spermatogenic maturation arrest and hypospermatogenesis.

## Introduction

Spermatogenesis is a well-regulated and hierarchical process to ensure prolonged male reproductive capability^1,2^. Sertoli cells (SCs) extend their thin cytoplasm arms around all the germ cells (GCs) to nurture their development and these intercellular associations are maintained throughout the process of spermatogenesis. Mature SCs form three types of intercellular junctions: cadherin-based adherens junctions, occludin-based tight junctions, and connexin-based gap junctions. These junctions are involved in forming the blood–testis barrier (BTB)^3^. The BTB divides seminiferous epithelium into the basal and apical (adluminal) compartments, where the different stages of spermatogenesis take place: the renewal and differentiation of spermatogonial stem cells (SSCs) and the preleptotene stage spermatocyte in the basal compartment, meiosis and spermiogenesis, and spermiation in the adluminal compartment^4^. In addition to BTB, the developing GCs also interact with SCs to form a number of distinct stage-specific junctions^5^. Thus the integrity and architecture of the SCs are critical for the orderly progression of spermatogenesis^6,7^.

In varicocele patients, dysfunction of spermatogenesis may be associated with BTB disruption^8^. Non-obstructive azoospermia (NOA) patients carried the WT1 mutant, and WT1 loss of function in mouse SCs led to BTB structural damage, which in turn resulted in GCs death^9^. According to previous report, undifferentiated spermatogonia was able to survive when the BTB was disrupted, and they were sufficient to restore spermatogenesis theoretically^10^. An in vitro study also demonstrated that SSCs isolated from obstructive azoospermia (OA) and NOA patients had high developmental capacity when supported with extracellular matrix (ECM) components^11^. These findings suggested the possibility that amelioration of disrupted BTB would restore spermatogenesis in NOA patients.

Based on our previous study of patients with male infertility who had been infected with the mumps virus before puberty, geranylgeranyl diphosphate synthase (*Ggpps*) deficiency in the SCs could induce excessive cytokine and chemokine synthesis, and result in the invasion of macrophage into seminiferous tubule during puberty when BTB was not completely formed. The macrophages in the seminiferous tubule would lead to the developing GCs death when they penetrated into the adluminal compartment and subsequently resulted in infertility in adult mice^12^. GGPPS is a branch point enzyme in the mevalonate pathway that catalyzes the synthesis of GGPP from farnesyl diphosphate (FPP)^13^, which can prenylate signaling proteins such as Ras family. Herein, we demonstrate that deletion of *Ggpps* in the SCs results in the destruction of the BTB and SC–GC adhesions through affecting the distribution of adhesion proteins in seminiferous tubules. Studies in *Drosophila* stem cells have shown that cadherins were indispensable for the stem cell–niche interaction^14^ and the downregulation of N-cadherin promoted germline stem cells (GSCs) differentiation by displacing GSCs away from the niche^15^, indicating that N-cadherin maintains the GSC pool. We speculated that this kind of regulation may be involved in maintaining the SSC pool in mammal.

In this study, we demonstrate that the integrity of BTB is critical for spermatogenesis because the structure not only seals the GCs from the immune system as previous report, but also determines the distinct interactions between the SCs and the GCs at different developmental stages. We also explore the possibility that berberine could restore spermatogenesis via resealing the damaged BTB and propose that amelioration of disrupted BTB may be an effective strategy for the treatment of male infertility.

## Materials and methods

### Study approval

Mice were housed according to mouse welfare and ethics of Nanjing University in groups with 12-h dark–light cycles and free access to food and water. The experimental animal facility has been accredited by Association for Assessment and Accreditation of Laboratory Animal Care International (AAALAC) and all animal protocols used in this study were approved by the Institutional Animal Care and Use Committee (IACUC) of Model Animal Research Center of Nanjing University. We collected 18 NOA patients and 5 OA patients, respectively, to perform immunofluorescence and immunohistochemistry staining and seven NOA patients and three OA patients, respectively, to perform qRT-PCR. We obtained patient consent and approval beforehand for the use of clinical samples, which were from Nanjing General Hospital and used for research purposes only. All the studies abide by the Declaration of Helsinki principles

**Table 1:** PCR templates and primers used for gene manipulation

Gene symbol	Forward primer (5′–3′)	Reverse primer (5′–3′)
*Cre*	GCGGTCTGGCAGTAAAAACTATC	AATTGTGTGTGGTAGGGGTA
*Loxp*	GTGAAACAGCATTGCTGTCACTT	AACTTGCTTCAGAACTGAGC
*mGGPPS*	TTCACCAACACCTGTAACTC	TTATTGACAAGCCCAGAGC
*hGGPPS*	TGGAGAAGACTCAAGAAACAG	TCAGCCAATGATTAAATGCC
*Cldn11*	ATGGTAGCCACTTGCCTTCAG	AGTTCGTCCATTTTTCGGCAG
*TJP1*	ACCACCAACCCGAGAAGAC	CAGGAGTCATGGACGCACA
*Cldn4*	GTCCTGGGAATCTCCTTGGC	TCTGTGCCGTGACGATGTTG

### Mice and tissues

We generated Sertoli cell-specific *Ggpps* deletion mice by crossing AMH-Cre transgenic mice^16^ with *Ggpps*^*fl/fl*^ mice. No significant difference of fertility and weight were observed among heterozygous and wild-type mice from the same litter. Therefore, we used the heterozygous as controls in the present study. The reproductive capacity was determined by mating one male with three C57BL/6 females as previously published^17^. Genotyping was conducted by using PCR (the primers for the PCR and the qRT-PCR analyses are indicated in Table S1). The sperm production was determined by dissecting epididymis in 1X PBS, then incubating at 37 °C for 0.5 h and counting the number of sperm under a microscope. The protocol for isolating primary SCs was performed as previously reported^18,19^. Testis were fixed in 4% paraformaldehyde and embedded in paraffin, sectioned (5 μm), and placed on slides for immunofluorescence, immunohistochemistry, and Tunel assay (Table 1).

### Histology, immunofluorescence, Tunel assays, and biotin permeability assay

Hematoxylin & eosin (H&E) staining was performed on testis sections of the control and knockout mice as described previously^20^. For immunohistochemistry and immunofluorescence staining, paraffin sections were de-paraffinized, rehydrated, and boiled in citrate buffer (pH 6.0) to retrieve antigens. Then, paraffin sections and frozen sections were permeabilized, blocked, and incubated with the indicated primary antibodies at 4 °C overnight. Subsequently, the sections were incubated with secondary antibodies for 1 h at room temperature. We analyzed the spermatogenesis progression using GC marker MVH^21^, SSC marker Plzf^22^, spermtocyte marker Sycp3^23^, and round spermatid marker acrosin^24^. A biotin assay for BTB integrity was performed as previously described^25^.

### mRNA and protein expression assays, immunoprecipitation

Total RNA was isolated from the testis and primary Sertoli cells using Trizol reagent (Takara), and the cDNA was synthesized with the PrimeScriptTM RT Master Mix (Takara) according to the manufacturer’s protocol. Quantitative PCR was performed with the SYBRTM Select Master Mix (Applied Biosystems) using the Applied Biosystems 7300 Real-Time PCR system. The relative mRNA level values were normalized to β-actin to calculate fold-changes in expression. To analyze protein expression, the cells or testis were washed in ice-cold PBS and harvested using RIPA buffer supplemented with protease inhibitors. The resulting supernatant fraction was homogenized in 1x SDS–PAGE sample buffer and boiled for 5 min at 99 °C. For the immunoblotting, proteins were separated on an SDS–PAGE gel and transferred to a polyvinylidene difluoride (PVDF) membrane. Membranes were blocked and incubated with the indicated primary antibody overnight at 4 °C. Bound primary antibodies were detected by HRP-conjugated secondary antibodies and a chemiluminescent substrate.

For immunoprecipitation, testis were extracted using IP buffer and the lysates were centrifuged at 12,000 × *g* for 15 min. The supernatant was incubated with the primary antibody RhoA and Cdc42 overnight at 4 °C. The immune complexes were immunoprecipitated using protein A/G agarose beads. After several washes, the samples were boiled and analyzed using western blot. The RhoA activity was determined by using the appropriate activation Assay Kit purchased from NewEast Biosciences.

### Cell culture

The isolation of the primary SCs was performed as previously described. SCs were cultured in DMEM/F12 medium containing 10% FBS with penicillin (100 U/ml) and streptomycin (100 mg/ml). The cells were maintained in a humidified atmosphere that contained 5% CO_2_ at 37 °C for 24 h. After incubation, the cells were treated with a hypotonic solution (20 mM Tris, pH 7.4) for 1 min to remove the spermatogenic cells adhered to the Sertoli cells. After a 24-h culture, the SCs were collected for RNA and protein extraction.

### Triton X-114 extraction of hydrophobic proteins

Hydrophobic and hydrophilic proteins were purified using Triton X-114 extraction to determine the membrane localization of the small GTPase. In brief, testis or primary Sertoli cell were homogenized in Triton X-114 lysis buffer and the lysates were centrifuged at 12,000 × *g* for 15 min at 4 °C. The supernatant was incubated at 37 °C for 5 min until the lysate became turbid and was centrifuged at 12,000 × *g* for 5 min at room temperature. The upper phase was an aqueous phase containing hydrophilic proteins (water-soluble small G protein), and the lower phase was a detergent phase containing hydrophobic proteins (lipid-soluble small G protein). The ratio of hydrophobic/hydrophilic proteins shows the altered membrane association of small G proteins.

### Statistical analysis

All data were presented as the mean ± s.e.m. Statistical comparisons were performed with unpaired two-tailed Student’s *t*-test. ANOVA and Student’s *t* tests were carried out in GraphPad Prism5. In all cases, statistical significance was indicated as *p < 0.05 or **p < 0.01.

## Results

### The BTB structure and the cell–cell adhesion are disrupted in the NOA patients

**Fig. 1:**
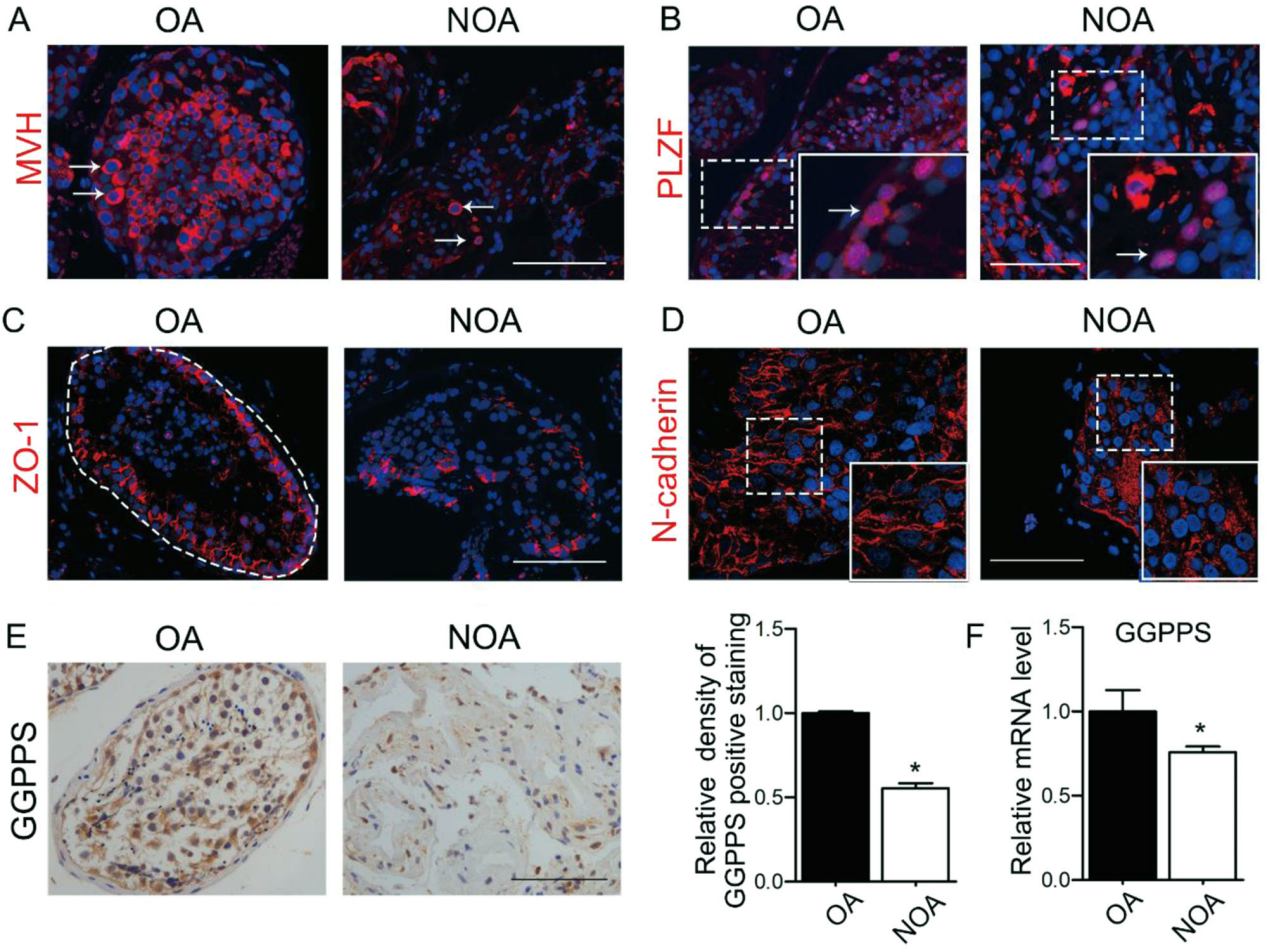
Disruption of the blood–testis barrier leads to spermatogenesis arrest in NOA patients. **a**, **b** Immunofluorescence of GC marker MVH and undifferentiated SSC marker Plzf in OA and NOA patients. The arrows indicate the positive staining. **c**, **d** Immunofluorescence staining of TJ-associated protein ZO-1 and adherens junction protein N-cadherin in OA and NOA patients. **e** Immunohistochemistry and density of the positive staining of GGPPS in OA and NOA patients. **f** Relative mRNA level of GGPPS in OA and NOA patients. *n* = 18 of NOA patients and *n* = 5 of OA patients to be performed for immunofluorescence and immunohistochemistry, respectively. *n* = 7 of NOA patients and *n* = 3 of OA patients to be performed for qRT-PCR, respectively. Data are presented as the mean ± SEM. *p < 0.05; **p < 0.01, *n* ≥ 3. Scale bar: 100 μm

There are different types of NOA: Sertoli cell only, maturation arrest, and hypospermatogenesis^26^, whose spermatogenesis is impeded and sperm production is largely disturbed (Fig. S1). We identified that the positive staining of GC marker MVH were able to be found in the basal compartment of NOA samples with both maturation arrest and hypospermatogenesis, but the number of the positive staining in NOA patients was decreased compared to the OA patients (Figs. 1a and S2A). These Plzf-positive staining cells in the basal compartment of the seminiferous epithelium in both the OA and NOA patients were undifferentiated spermatogonia, and the number of the Plzf-positive staining was similar in OA and NOA patients (Figs. 1b and S2B). To determine how the cellular junctions in the OA and NOA patients were situated, we investigated the distribution of ZO-1, a component of the BTB, and the distribution of N-cadherin responsible for adhesion between the SCs and GCs. We found that the structure of the BTB was incomplete in the seminiferous tubule of the NOA patients, while it was intact in the OA patients (Fig. 1c). The N-cadherin distribution in the OA patients was orderly on the cell surface of GCs but in the NOA patients was disorganized (Fig. 1d). Our studies suggested that the BTB and the SC–GC adhesions were disrupted, which might lead to spermatogenesis arrest in the NOA patients.

We previously reported that the oocyte-granulosa communication was blocked after *Ggpps* deletion in oocyte and the membrane location of cell junction proteins in oocyte was destroyed in knockout mice^27^. Thus, we determined the expression of GGPPS in testicular biopsy specimens of the NOA patients and found that mRNA level of GGPPS was decreased in the biopsy of NOA patients with maturation arrest and hypospermatogenesis. In addition, the immunohistochemistry staining showed that the GGPPS-positive staining was deceased in NOA patients compared to that in OA patients (Fig. 1e, f). So, we hypothesized that the decreased GGPPS in the SCs might be responsible for spermatogenesis arrest in the NOA patients with maturation arrest and hypospermatogenesis via disrupting the cell–cell junction, especially the BTB.

### *Ggpps* deletion in SCs results in spermatocyte loss in the adluminal compartment and does not affect spermatogonia in the basal compartment

**Fig. 2:**
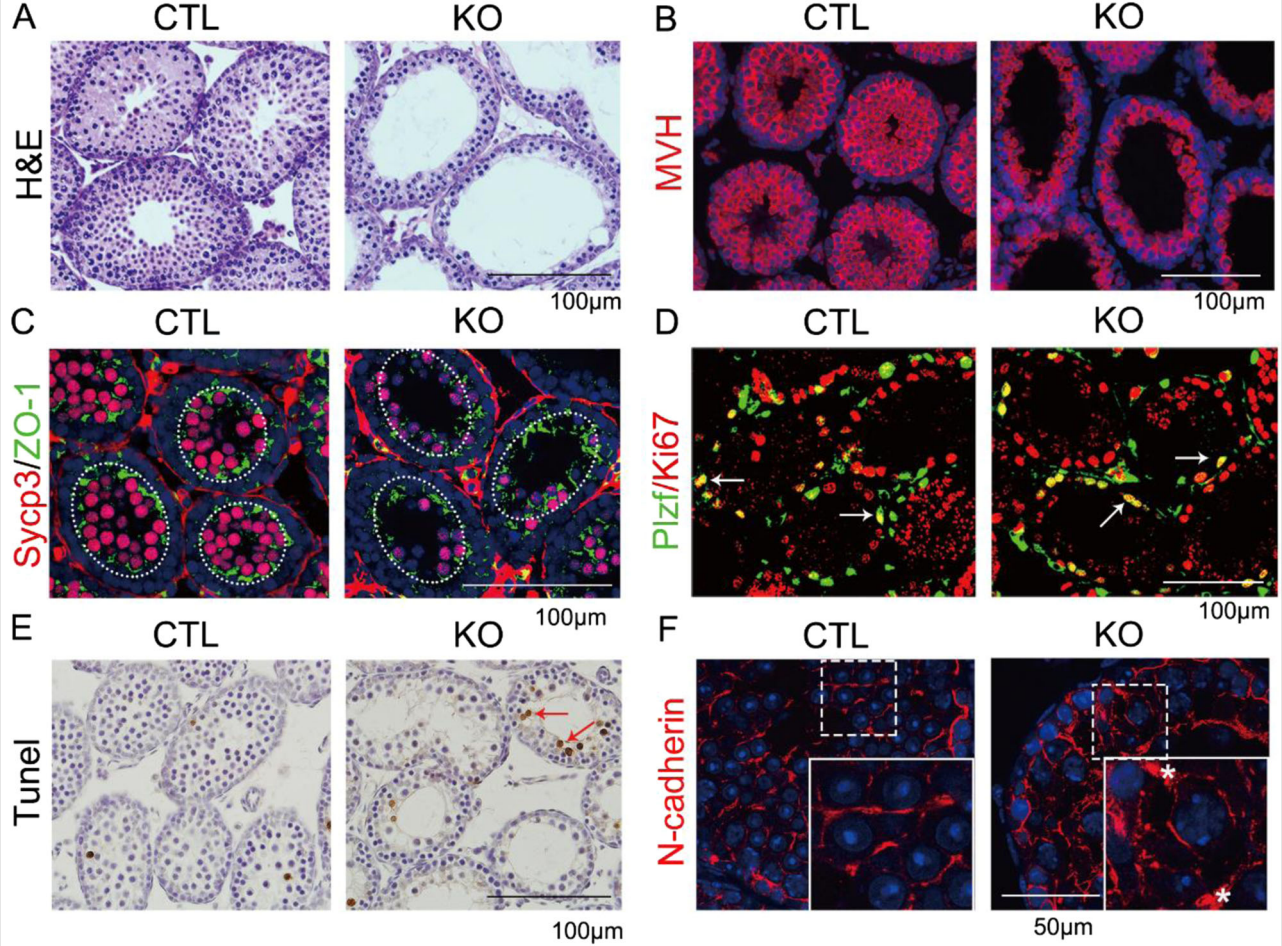
*Ggpps* deletion results in GC loss in the adluminal compartment while not in the basal compartment. **a** H&E staining of mice testis in CTL and KO mice at 3 weeks old. **b** Immunofluorescence of GC marker MVH in CTL and KO mice testis at 3 weeks old. **c** Co-immunofluorescence of spermatocyte marker Sycp3 and TJ-associated protein ZO-1 at 2 weeks old. **d** Co-immunofluorescence of the undifferentiated SSC marker Plzf and proliferation marker Ki67 at 2 weeks old. The white arrow indicates the co-staining of Plzf and Ki67. **e** Tunel assay in CTL and KO mice testis. The red arrow indicates the Tunel-positive staining. **f** Immunofluorescence of adherens junction protein n-cadherin in CTL and KO mice testis. The asterisk indicates the distribution of N-cadherin in apical cytoplasm of SCs. *n* ≥ 3

We deleted *Ggpps* in SCs by crossing the *Ggpps*-floxed mice with Amh-Cre transgenic mice^28^, which expressed Cre recombinase from E13.5 day specifically in SCs. We chose the 2 and 3 weeks old mice during the first wave of spermatogenesis for experiments to exclude other complicated reasons causing the phenotype after 4 weeks old. H&E staining showed most of seminiferous tubules had few spermatocytes in the SC-*Ggpps*^−/−^ mice, whereas multilayered spermatogenic cells filled the seminiferous tubules in the WT mice (Fig. 2a). The GC marker MVH staining indicated that *Ggpps* deletion resulted in a striking loss of spermatocytes (Fig. 2b). The double staining of the BTB-associated protein ZO-1 and spermatocyte marker Sycp3 further showed that the spermatocyte number was largely decreased in the adluminal compartment above the BTB (Figs. 2c and S3A). Meanwhile, surviving SSCs were observed close to the basal compartment in SC-*Ggpps*^−/−^ mice (Fig. 2b). The GCs retained in the basal compartment were undifferentiated SSCs, which were confirmed by the immunostaining of the SSC marker Plzf (Figs. 2d and S3B). Furthermore, double staining of Plzf and proliferation marker Ki67 suggested that SSCs were still capable of self-renewal (Figs. 2d and S3C). Other than the 3-week-old mice, in older mice, such as 16-week-old knockout mice, we also found that the number of germ cells was less than that in WT mice (Fig. S3D). Furthermore, the Tunel assay also indicated that the spermatocyte underwent apoptosis in the adluminal compartment (Figs. 2e and S3E). These data suggested that the spermatogenesis was blocked and spermatocytes ceased development. N-cadherin staining showed that the organization of the SC–GC adhesions was disrupted and N-cadherin was accumulated in the apical cytoplasm of SCs in SC-*Ggpps*^−/−^ mice testis (Fig. 2f). The results disclosed that *Ggpps* deletion in SCs resulted in spermatogenesis arrest and loss of spermatocyte just like the NOA patients with maturation arrest and hypospermatogenesis did, which was also associated with cell adhesion disorganization (Fig. 1c, d).

### *Ggpps* deletion in SC leads to the destruction of the BTB and spermatocyte–SC adhesion

**Fig. 3:**
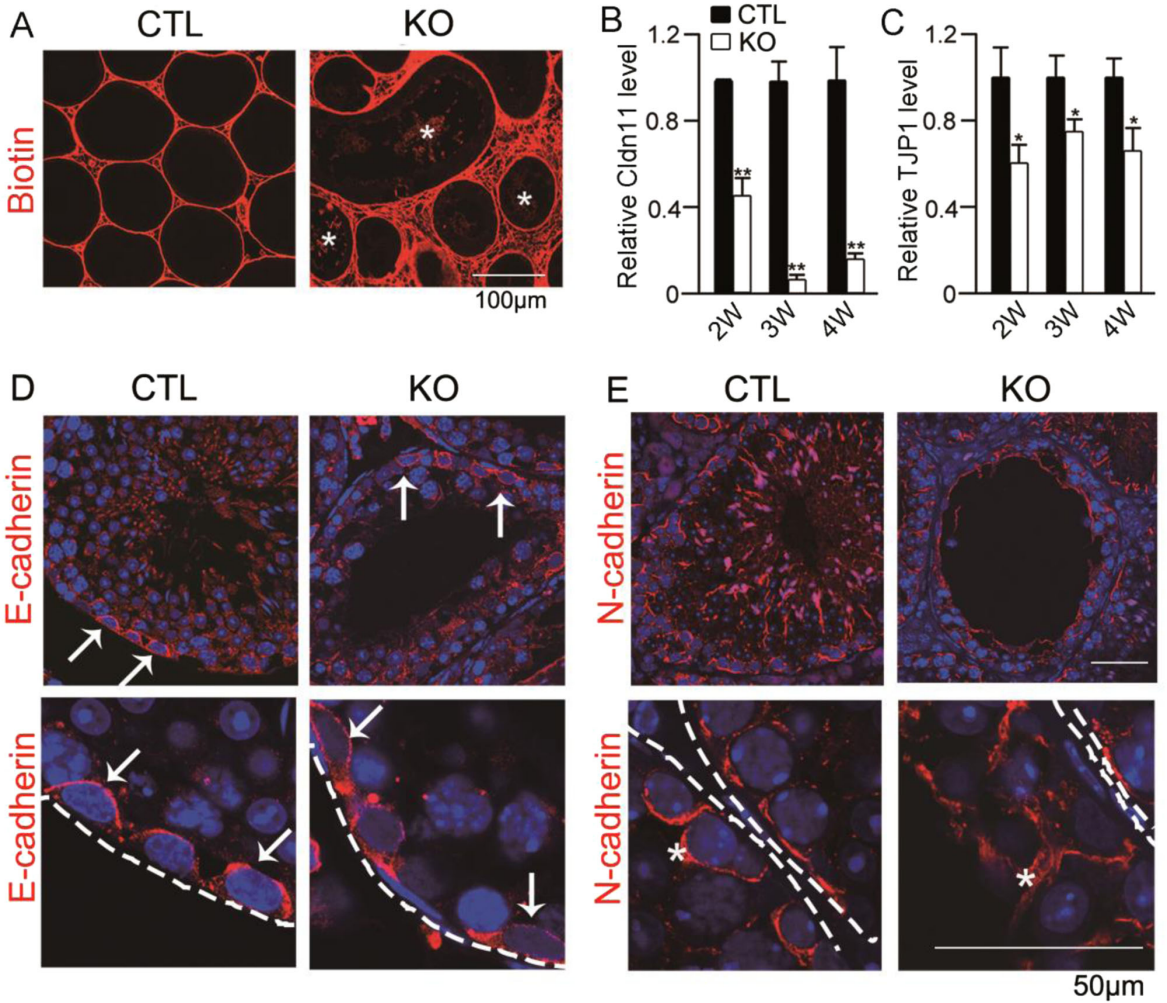
*Ggpps* deletion in SC leads to the destruction of BTB and spermatocyte–SC adhesion. **a** Biotin distribution in CTL and KO mice testis after biotin injection at 4 weeks old. The asterisk indicates the biotin penetrating into seminiferous tubules. **b**, **c** RT-qPCR analysis of tight junction protein: claudin11 and TJP1 during BTB establishment. **d**, **e** Immunofluorescence of adherens junction protein E-cadherin and N-cadherin in CTL and KO mice testis. The dashed line indicates the bottom of seminiferous tubules. The white arrow indicates the distribution of E-cadherin and N-cadherin in testis. Data are presented as the mean ± SEM. *p < 0.05, **p < 0.01, *n* ≥ 3

In our previous report, we noted that *Ggpps* deletion in SCs resulted in macrophage invasion into seminiferous tubules, and the infertile phenotype of the SC-*Ggpps*^−/−^ mice was similar to the characterization of azoospermia^12^. BTB was established from 2-week old and accomplished at nearly 4 weeks old, which was formed by the interaction of adjacent SCs^29^. When mice testis were injected with fluorescent-labeled biotin, the biotin was able to permeate into seminiferous tubule in SC-*Ggpps*^−/−^ mice due to the degenerated BTB (Fig. 3a). We had isolated SCs from 3-day-old mice testis and analyzed the gene expression with microarray (GSE35755)^12^. The data showed that the cell junction-associated protein expression was decreased in *Ggpps*^−/−^ mice (Fig. S4). This decrease was sustained as long as 4 weeks until adulthood (Fig. 3b, c). It is reported that N-cadherin mediates the adhesions between all spermatogenic cells and SCs^30^, while E-cadherin only mediates the interaction of SSCs and SCs in mammals^31^.

We found that the E-cadherin was exclusively located in the basal compartment, and was not altered after *Ggpps* deletion (Fig. 3d). Unlike E-cadherin, N-cadherin was located in the basal compartment and the apical ectoplasmic specialization in the adluminal compartment^32^. *Ggpps* deletion resulted in the dispersion of N-cadherin into the cytoplasm of SCs (Fig. 3e). Thus, we speculated that the arrested spermatogenesis in SC-*Ggpps*^−/−^ mice was associated with the defective BTB and spermatocyte–SC adhesions.

### *Ggpps* affects the BTB and cell adhesion via regulating protein isoprenylation of small G-protein

**Fig. 4:**
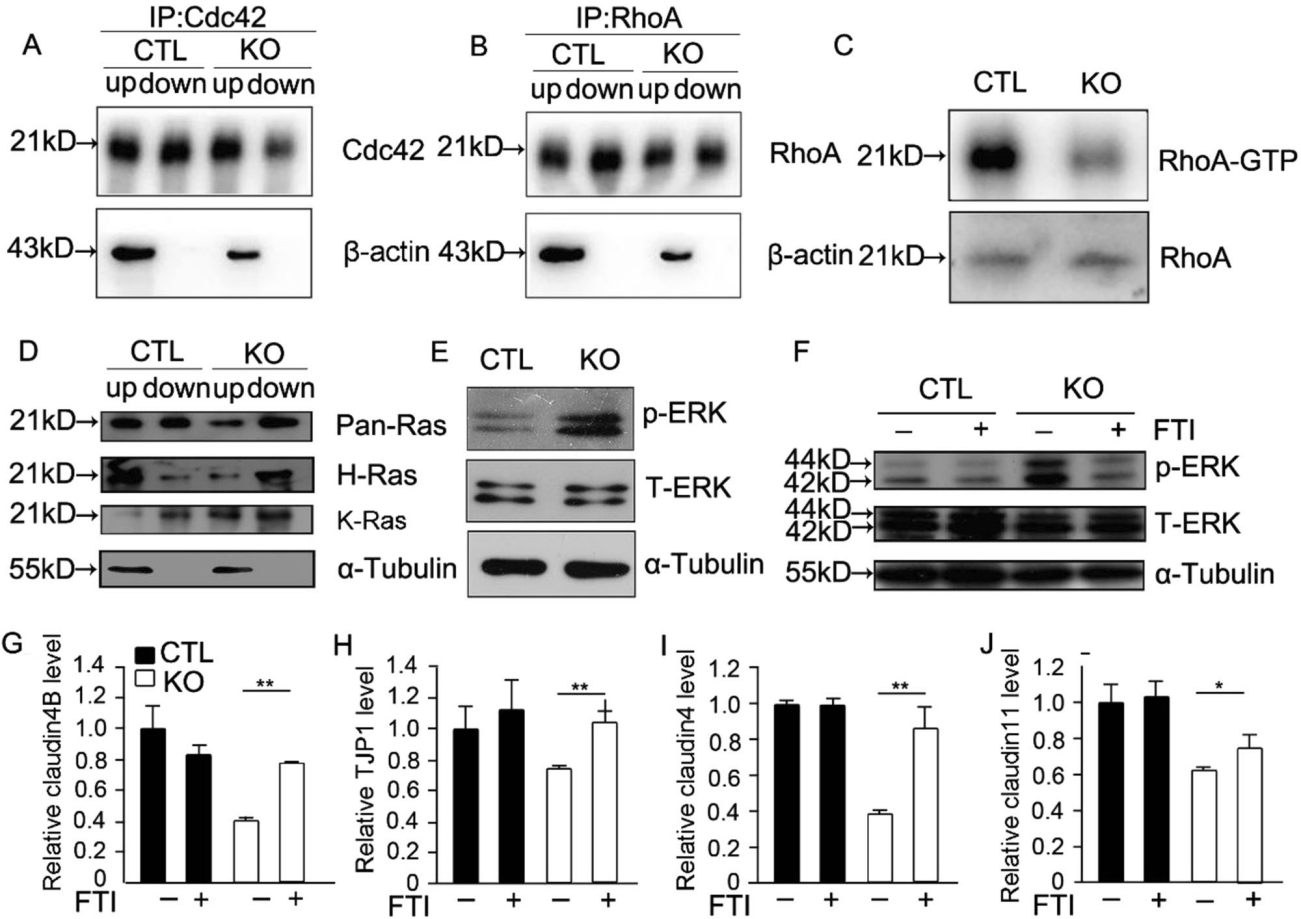
*Ggpps* affects the BTB and cell adhesion via the regulation of protein isoprenylation of small G-protein. **a**, **b** Immunoblot with Rho family members Cdc42 and RhoA of hydrophobic proteins (down) and hydrophilic proteins (up) in CTL and KO mice testis. **c** Immunoblot with the active RhoA form RhoA-GTP in CTL and KO mice testis. **d** Immunoblot with Pan-Ras, H-Ras, and K-Ras in hydrophobic proteins (down) and hydrophilic (up) proteins of primary SC. **e** Immunoblot with p-ERK and T-ERK of primary SC in control and KO mice relative to α-tubulin. **f** Immunoblot with p-ERK and T-ERK after FTI treatment of primary SC in control and KO mice relative to α-tubulin. **g**–**j** Relative mRNA level of tight junction proteins after FTI treatment. Data are presented as the mean ± SEM. *p < 0.05; **p < 0.01, *n* ≥ 3

We have found that the balance of protein farnesylation and geranylgeranylation was critical for cell function^12,27,33,34^. *Ggpps* deletion leads to FPP accumulation and GGPP decline, which enhances protein farnesylation and decreases protein geranylgeranylation. For example, FPP accumulation would increase farnesylation of Ras^12^ and GGPP decline would decrease geranylgeranylation of the Rab^33^ and Rho family^27^. Cell adhesion and actin cytoskeleton can be regulated by Rho family^35,36^. Then, we isolated the hydrophilic and hydrophobic proteins by tritonX-114 extraction and found that the hydrophobic Cdc42 and RhoA (membrane bound form) was decreased, which suggested that the geranylgeranylation of Cdc42 and RhoA was inhibited after *Ggpps* deletion (Figs. 4a, b and S5A and B). In addition, RhoA GTP activity was also decreased although without significant difference due to the sample variation (Figs. 4c and S5C). These data suggested that the disrupted SC–GC adhesions after *Ggpps* deletion was attributed to the defected protein geranylgeranylation of Rho family.

We also found that the *Ggpps* deletion largely led to an increase in the farnesylation level of Ras in primary SC (Fig. 4d and S5D). The increased membrane association of Ras in SC-*Ggpps*^−/−^ mice resulted in the activation of extracellular signal-regulated kinase 1/2 (Erk1/2) signaling (Fig. 4e), which could be blocked by FTI, an inhibitor of farnesyltranferase and the FTI treatment concentration we used was 10 μM^37^ (Fig. 4f). As to other mitogen-activated protein kinases (MAPK) family members: p38 and Jnk, we detected their phosphorylation and total level of p38 and Jnk, and the results demonstrated that the phosphorylation level of p38 and Jnk was not changed in KO mice. Previous studies demonstrated that ERK and p38 had diverse biological functions^38^ and they could regulate apoptosis in an opposing manner^39^, which explained that the different changes between ERK and p38 in our study (Fig. S5E and F). Furthermore, inhibition of protein farnesylation could increase the expression of BTB components, such as claudin 4, claudin 4B, claudin 1, and tight junction protein 1 (TJP1) in SC-*Ggpps*^−/−^ SCs (Fig. 4g–j). The results indicated that enhancement of Ras farnesylation might be related with the BTB destruction through decreasing the expression of BTB-associated proteins.

### Berberine can partially alleviate arrested spermatogenesis in SC*-Ggpps*^−/−^ mice testis

**Fig. 5:**
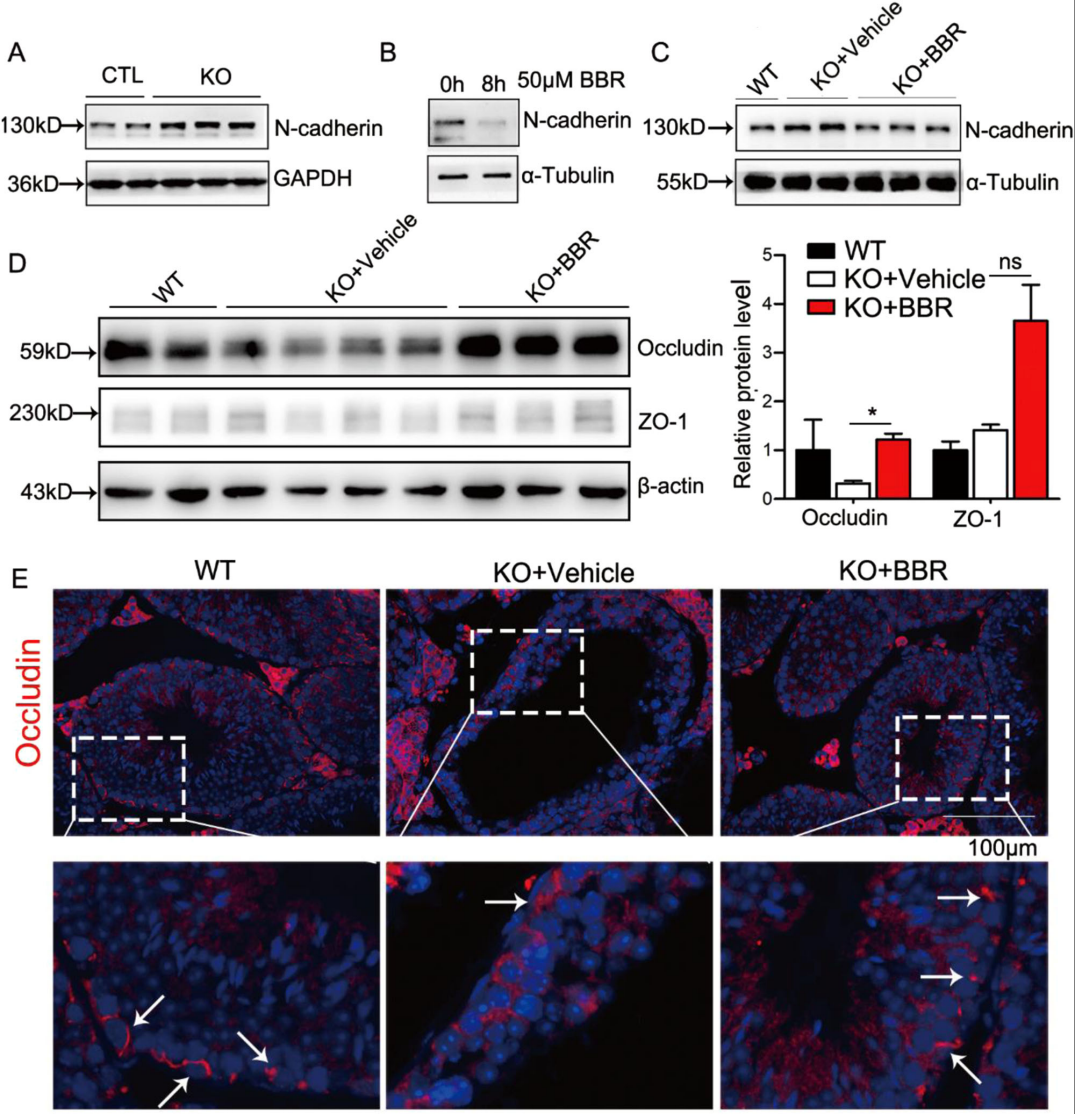
Berberine treatment could ameliorate the damaged tight junction. **a** Immunoblot with n-cadherin in CTL and KO mice testis relative to GAPDH. **b** Immunoblot with N-cadherin of primary SC in KO mice after berberine treatment relative to α-Tubulin. **c** Immunoblot with N-cadherin in WT and KO mice testis after berberine treatment relative to α-tubulin. **d** Immunoblot and statistical analysis of tight junction proteins occludin and ZO-1 in WT and KO mice testis after berberine treatment relative to β-actin. **e** Immunofluorescence with occludin in WT and KO mice testis after berberine treatment. Data are presented as the mean ± SEM. *p < 0.05; **p < 0.01, *n* ≥ 3. Scale bar: 100 μm

Our above studies indicated that the destructed BTB and cell adhesions led to spermatogenesis defect in NOA patients, so we tried to seek methods to restore the BTB structure to regenerate spermatogenesis. Berberine, a natural product isolated from Chinese herb, has been used in traditional Eastern medicine for a long time to treat gastroenteritis, abdominal pain, and diarrhea^40–42^. Previous studies of berberine function focused on its regulation of cholesterol-lowering^43^, antidiabetic^44^, and potential role in restoring broken down barrier in intestinal inflammation^45^. Berberine prevents TNFα-induced claudin-1 disassembly and upregulates claudin-2 at its mRNA level^45^. Here, *Ggpps* deletion induced an increase in N-cadherin expression (Figs. 5a and S6A), which was accumulated in cytoplasm and could not be assembled into cell adhesion complex on the cell membrane (Fig. 3f). Berberine treatment could effectively reduce the elevated N-cadherin level caused by *Ggpps* deletion in isolated primary SCs (Figs. 5b and S6B). The results encouraged us to investigate whether berberine could ameliorate the cell adhesions to restore spermatogenesis.

Then we treated the 4 weeks old SC-*Ggpps*^−/−^ mice with berberine by oral gavage at a dose of 200 mg/kg/day. After 5 weeks, we sacrificed the mice and found that the expression of N-cadherin was decreased significantly (Figs. 5c and S6C), suggesting that berberine treatment may promote spermatogenesis recovery by improving the damaged adhesion junction. Furthermore, we detected the expression level of tight junction proteins and the BTB structure in mice testis after berberine treatment. The expression level of tight junction protein occludin in mice testis was increased after berberine treatment (Fig. 5d). Also the expression level of tight junction associated protein ZO-1 was increased, but there is no significant difference because of the sample variation between different mice testis (Fig. 5d). Occludin in WT mice was located between the SCs along the BTB at the basal compartment, but the distribution of occludin was dispersed into the cytoplasm in SC-*Ggpps*^−/−^ mice. According to our hypothesis the berberine treatment could ameliorate the disrupted BTB, and also the cytosolic dispersed occludin in SC-*Ggpps*^−/−^ mice was relocated between the SCs along the BTB at the basal compartment after this treatment (Fig. 5e). These data revealed that berberine could ameliorate the disrupted BTB to some extent.

**Fig. 6:**
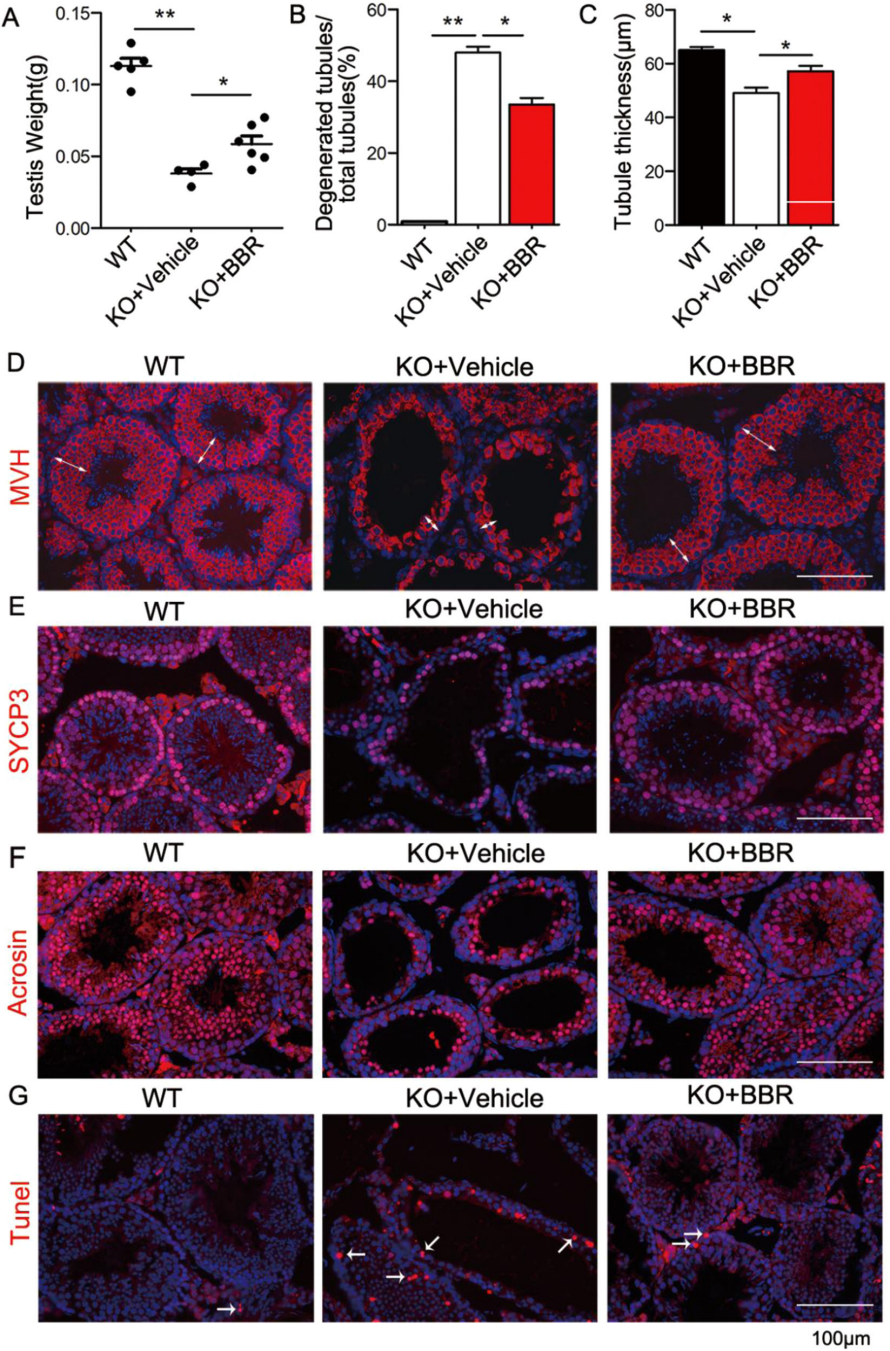
Berberine restores spermatogenesis via ameliorating the damaged tight junction. **a**, **b** Analysis of testis weights and degenerated tubules in KO mice testis after berberine treatment relative to WT mice. **c**, **d** The epithelium thickness and immunofluorescence with GC marker MVH in WT and KO mice testis after berberine treatment. White double arrow indicates the epithelium thickness. **e**, **f** Immunofluorescence with spermatocyte marker Sycp3 and round spermatid marker acrosin in WT and KO mice after berberine treatment. **g** Tunel assay in WT and KO mice after berberine treatment. Data are presented as the mean ± SEM. *p < 0.05, **p < 0.01, *n* ≥ 3

We determined the sperm production and found that the sperm number was elevated after berberine treatment (Fig. S6D). Further analysis showed that berberine treatment could effectively increase the testis weight and restore the degenerated tubules in SC-*Ggpps*^−/−^ mice (Fig. 6a, b). The thickness of the seminiferous tubule was measured in accordance with the semi-diameter of seminiferous tubule minus the semi-diameter of luminal tubule. The immunofluorescence of the GC marker MVH showed that berberine could increase the seminiferous epithelium thickness (Fig. 6c, d). To figure out whether meiosis was resumed after berberine treatment, we detected the immunofluorescence of spermatocyte marker Sycp3 and found that the positive staining cells were increased in KO mice after berberine treatment (Figs. 6e and S6E). Also, we found that spermatocytes could develope into round spermatids, because the positive staining of the round spermatid marker acrosin were increased in KO mice after berberine treatment (Figs. 6f and S6E). These evidences suggested that meiosis was resumed. In addition, the Tunel assay showed that berberine treatment could decrease the apoptosis in KO mice (Figs. 6g and S6F). Our study indicated that berberine administration would be an effective strategy for NOA therapy.

## Discussion

Currently, there is no effective therapeutic strategy for azoospermia or oligozoospermia, especially for the NOA patients. Our study indicated that BTB and cell adhesions were disrupted in the NOA patients. Recent reports suggest that by resealing the toxicant-disrupted BTB, spermatogenesis can be restored in the mouse model^46^. Here, we showed that the broken BTB blocked spermatocyte development in the adluminal compartment. But the SSCs in the basal compartment under the BTB survived, which made it possible for us to restore spermatogenesis through the SC function recovery, especially BTB reconstruction. We showed that berberine may be an effective therapeutic strategy to reinitiate spermatogenesis in NOA patients with maturation arrest and hypospermatogenesis through ameliorating disrupted BTB, which were demonstrated in a SC-*Ggpps*^−/−^ mouse model.

In this study, we generated a mouse model by deleting *Ggpps* in SCs, which could result in the disruption of BTB by breaking the balance of protein farnesylation and geranylgeranylation. In addition to the damaged BTB, the SC-*Ggpps*^−/−^ mice also showed the disruption of SC–GC adhesions, which closely resembled the observation in NOA patients. The similar characteristics of the seminiferous tubules in SC-*Ggpps*^−/−^ mice and the NOA patients made it convenient to investigate the mechanism of azoospermia or oligozoospermia directly and effectively. Here, we focused on how to restore the spermatogenesis in NOA patients by using this animal model and found that the reconstruction of BTB and cell adhesions was a promising strategy for treating azoospermia or oligozoospermia.

In *Drosophila* testis, the occludin junction and gap junction are of particular importance in maintaining SSC niche homeostasis^47,48^. Occludin junctions shape the signaling environment between the somatic cells and the germ cells to maintain niche homeostasis. SSC niche homeostasis is likely regulated by the occluding junction in mammal in similar manner. The BTB separates the seminiferous tubule into two parts: basal and adluminal compartments. The adhesion proteins on the cell surface of the SC and spermatogenic cell seem to be specified by the BTB^30,31^. E-cadherin is specially distributed on SSC in the basal compartment, whereas N-cadherin is distributed in the basal compartment and the apical ectoplasmic specialization in the adluminal compartments^32^. The different types of SC–GC adhesion are probably engaged in different “physical” niche microenvironment in the basal and adluminal compartments. Therefore, the BTB is an important structure that contributes to the survival of SSC and development of the spermatocytes and spermatids by defining distinct “chemical” and “physical” niches in the basal and adluminal compartments, respectively. When berberine was used to treat the SC-*Ggpps*^−/−^ mice, the “physical” niche was recovered via regulating N-cadherin expression.

Protein prenylation is critical not only for targeting proteins to cellular membranes but also in protein–protein interactions^49,50^. And the functional role of protein prenylation has been investigated for Ras and Rho family. The prenylation of Rho family member RhoA is involved in cytoskeleton organization, which maintains the actin stress fiber content and focal adhesions^51^. Rho GTPases are post-translationally modified by prenylation. In addition, Rho family GTPases are involved in the regulation of cadherin-mediated cell–cell adhesion and cytoskeleton. The GTP-bound active forms of Cdc42 and Rac1 interact with their downstream target IQGAP1 and thereby prohibit IQGAP1 from interacting with β-catenin, leading to the interaction of E-cadherin and β-catenin, which establish the strong adhesion^52,53^. Statin, a cholesterol-lowering agent that inhibit 3-hydroxy-3-methylglutaryl-coenzyme A (HMG-CoA) reductase, reduces the membrane localization of K-Ras and down regulates the testosterone level^35,54^, which can promote BTB assembly by accelerating the kinetics of endocytosis and recycling of BTB-associated integral membrane proteins, including occludin, junctional adhesion molecule A (JAM-A), and N-cadherin^55^. Statin can also down regulate the inflammation level^56^. The evidence suggested that the regulation of mevalonate metabolic pathway may be a reasonable target for regenerating spermatogenesis.

In summary, we found that *Ggpps* deletion resulted in the BTB destruction in males, and our further investigation determined that berberine could improve the damaged adhesion junction-mediated BTB to reinitiate spermatogenesis. These results suggested that resealing BTB could be an effective therapeutic target for male infertility.

## Supplementary information

Supplementary figures S1-S6 and supplemental tables 1-2

## References

[1] Nakagawa T, Nabeshima Y, Yoshida S (2007). Functional identification of the actual and potential stem cell compartments in mouse spermatogenesis. Dev. Cell.

[2] Oatley JM, Brinster RL (2012). The germline stem cell niche unit in mammalian testes. Physiol. Rev..

[3] Dym M, Fawcett DW (1970). The blood-testis barrier in the rat and the physiological compartmentation of the seminiferous epithelium. Biol. Reprod..

[4] Cheng CY, Mruk DD (2012). The blood–testis barrier and its implications for male contraception. Pharmacol Rev..

[5] Enders GC, Millette CF (1988). Pachytene spermatocyte and round spermatid binding to Sertoli cells in vitro. J. Cell Sci..

[6] Wu D (2017). SENP3 grants tight junction integrity and cytoskeleton architecture in mouse Sertoli cells. Oncotarget.

[7] Wen Q (2018). Actin nucleator Spire 1 is a regulator of ectoplasmic specialization in the testis. Cell Death Dis..

[8] Koksal IT (2007). Varicocele-induced testicular dysfunction may be associated with disruption of blood-testis barrier. Arch. Androl..

[9] Wang XN (2013). The Wilms tumor gene, Wt1, is critical for mouse spermatogenesis via regulation of sertoli cell polarity and is associated with non-obstructive azoospermia in humans. PLoS Genet..

[10] van der Heijden GW (2007). Chromosome-wide nucleosome replacement and H3.3 incorporation during mammalian meiotic sex chromosome inactivation. Nat Genet..

[11] Lim JJ (2010). Long-term proliferation and characterization of human spermatogonial stem cells obtained from obstructive and non-obstructive azoospermia under exogenous feeder-free culture conditions. Cell Prolif..

[12] Wang XX (2013). Altered protein prenylation in Sertoli cells is associated with adult infertility resulting from childhood mumps infection. J. Exp. Med..

[13] Goldstein JL, Brown MS (1990). Regulation of the mevalonate pathway. Nature.

[14] Losick VP, Morris LX, Fox DT, Spradling A (2011). Drosophila stem cell niches: a decade of discovery suggests a unified view of stem cell regulation. Dev. Cell.

[15] Epstein Y (2017). miR-9a modulates maintenance and ageing of Drosophila germline stem cells by limiting N-cadherin expression. Nat. Commun..

[16] Lecureuil C, Fontaine I, Crepieux P, Guillou F (2002). Sertoli and granulosa cell-specific Cre recombinase activity in transgenic mice. Genesis.

[17] Chang C (2004). Infertility with defective spermatogenesis and hypotestosteronemia in male mice lacking the androgen receptor in Sertoli cells. Proc. Natl Acad. Sci. USA.

[18] Wang H (2006). Evaluation on the phagocytosis of apoptotic spermatogenic cells by Sertoli cells in vitro through detecting lipid droplet formation by Oil Red O staining. Reproduction.

[19] Sun B (2010). Sertoli cell-initiated testicular innate immune response through toll-like receptor-3 activation is negatively regulated by Tyro3, Axl, and mer receptors. Endocrinology.

[20] Fischer A. H., Jacobson K. A., Rose J., Zeller R. (2008). Hematoxylin and Eosin Staining of Tissue and Cell Sections. Cold Spring Harbor Protocols.

[21] Tanaka SS (2000). The mouse homolog of Drosophila Vasa is required for the development of male germ cells. Genes Dev..

[22] Buaas FW (2004). Plzf is required in adult male germ cells for stem cell self-renewal. Nat. Genet..

[23] Yuan L (2000). The murine SCP3 gene is required for synaptonemal complex assembly, chromosome synapsis, and male fertility. Mol. Cell.

[24] Klemm U, Muller-Esterl W, Engel W (1991). Acrosin, the peculiar sperm-specific serine protease. Hum. Genet..

[25] Meng J, Holdcraft RW, Shima JE, Griswold MD, Braun RE (2005). Androgens regulate the permeability of the blood-testis barrier. Proc. Nat Acad. Sci. USA.

[26] Ramasamy R, Yagan N, Schlegel PN (2005). Structural and functional changes to the testis after conventional versus microdissection testicular sperm extraction. Urology.

[27] Jiang C (2017). GGPP-mediated protein geranylgeranylation in oocyte is essential for the establishment of oocyte-granulosa cell communication and primary-secondary follicle transition in mouse ovary. PLoS Genet..

[28] Holdcraft RW, Braun RE (2004). Androgen receptor function is required in Sertoli cells for the terminal differentiation of haploid spermatids. Development.

[29] Setchell BP (2008). Blood-testis barrier, junctional and transport proteins and spermatogenesis. Adv. Exp. Med. Biol..

[30] Newton SC, Blaschuk OW, Millette CF (1993). N-cadherin mediates Sertoli cell-spermatogenic cell adhesion. Dev. Dyn..

[31] Tokuda M, Kadokawa Y, Kurahashi H, Marunouchi T (2007). CDH1 is a specific marker for undifferentiated spermatogonia in mouse testes. Biol. Reprod..

[32] Lee NP, Mruk DD, Conway AM, Cheng CY (2004). Zyxin, axin, and Wiskott-Aldrich syndrome protein are adaptors that link the cadherin/catenin protein complex to the cytoskeleton at adherens junctions in the seminiferous epithelium of the rat testis. J. Androl..

[33] Jiang S (2016). GGPPS-mediated Rab27A geranylgeranylation regulates beta cell dysfunction during type 2 diabetes development by affecting insulin granule docked pool formation. J. Pathol..

[34] Diao F (2016). Alteration of protein prenylation promotes spermatogonial differentiation and exhausts spermatogonial stem cells in newborn mice. Sci. Rep..

[35] Tsubaki M (2011). Blockade of the Ras/MEK/ERK and Ras/PI3K/Akt pathways by statins reduces the expression of bFGF, HGF, and TGF-beta as angiogenic factors in mouse osteosarcoma. Cytokine.

[36] Lerner EC (1995). Ras CAAX peptidomimetic FTI-277 selectively blocks oncogenic Ras signaling by inducing cytoplasmic accumulation of inactive Ras-Raf complexes. J. Biol. Chem..

[37] Pille JY (2005). Anti-RhoA and anti-RhoC siRNAs inhibit the proliferation and invasiveness of MDA-MB-231 breast cancer cells in vitro and in vivo. Mol. Ther..

[38] Roux PP, Blenis J (2004). ERK and p38 MAPK-activated protein kinases: a family of protein kinases with diverse biological functions. Microbiol. Mol. Biol. Rev..

[39] Xia Z, Dickens M, Raingeaud J, Davis RJ, Greenberg ME (1995). Opposing effects of ERK and JNK-p38 MAP kinases on apoptosis. Science.

[40] Zhou Y (2015). Berberine prevents nitric oxide-induced rat chondrocyte apoptosis and cartilage degeneration in a rat osteoarthritis model via AMPK and p38 MAPK signaling. Apoptosis.

[41] Shirwaikar A, Shirwaikar A, Rajendran K, Punitha IS (2006). In vitro antioxidant studies on the benzyl tetra isoquinoline alkaloid berberine. Biol. Pharm. Bull..

[42] Baron ED, Kirkland EB, Domingo DS (2008). Advances in photoprotection. Dermatol. Nurs..

[43] Kong W (2004). Berberine is a novel cholesterol-lowering drug working through a unique mechanism distinct from statins. Nat. Med..

[44] Lee YS (2006). Berberine, a natural plant product, activates AMP-activated protein kinase with beneficial metabolic effects in diabetic and insulin-resistant states. Diabetes.

[45] Amasheh M (2010). TNFalpha-induced and berberine-antagonized tight junction barrier impairment via tyrosine kinase, Akt and NFkappaB signaling. J. Cell Sci..

[46] Li N (2016). Connexin 43 reboots meiosis and reseals blood-testis barrier following toxicant-mediated aspermatogenesis and barrier disruption. FASEB J..

[47] Fairchild MJ, Yang L, Goodwin K, Tanentzapf G (2016). Occluding junctions maintain stem cell niche homeostasis in the fly testes. Curr. Biol..

[48] Smendziuk CM, Messenberg A, Vogl AW, Tanentzapf G (2015). Bi-directional gap junction-mediated soma-germline communication is essential for spermatogenesis. Development.

[49] Marshall CJ (1993). Protein prenylation: a mediator of protein–protein interactions. Science.

[50] Zhang FL, Casey PJ (1996). Protein prenylation: molecular mechanisms and functional consequences. Annu. Rev. Biochem..

[51] Allal C (2000). RhoA prenylation is required for promotion of cell growth and transformation and cytoskeleton organization but not for induction of serum response element transcription. J. Biol. Chem..

[52] Kaibuchi K, Kuroda S, Fukata M, Nakagawa M (1999). Regulation of cadherin-mediated cell-cell adhesion by the Rho family GTPases. Curr. Opin. Cell Biol..

[53] Ren XD, Kiosses WB, Schwartz MA (1999). Regulation of the small GTP-binding protein Rho by cell adhesion and the cytoskeleton. EMBO J..

[54] Corona G (2010). The effect of statin therapy on testosterone levels in subjects consulting for erectile dysfunction. J. Sex. Med..

[55] Yan HH, Mruk DD, Lee WM, Cheng CY (2008). Blood-testis barrier dynamics are regulated by testosterone and cytokines via their differential effects on the kinetics of protein endocytosis and recycling in Sertoli cells. FASEB J..

[56] St Sauver JL (2011). Statin use and decreased risk of benign prostatic enlargement and lower urinary tract symptoms. BJU Int..

