# Supplementary material for: The alteration of RhoA geranylgeranylation and Ras farnesylation breaks the integrity of the blood–testis barrier and results in hypospermatogenesis
Source: Cell Death Dis. 2019 Jun 6;10(6):450. doi: 10.1038/s41419-019-1688-9 (PMC6554403; doi:10.1038/s41419-019-1688-9)
Supplement: Supplementary file 1 — Supplementary figures S1-S6 and supplemental tables 1-2 [file 41419_2019_1688_MOESM1_ESM.pdf]

The alteration of RhoA geranylgeranylation and Ras farnesylation breaks the integrity of the blood-testis barrier and results in hypospermatogenesis

Ruilou Zhu[1,4], Jiangnan Wang[1], Tianxiang Feng[1,4], Xuechun Hu[2], Chen Jiang[1], Xiuxing Wang[1], Kang Li[1,4], Yongjuan Sang[1,4], Yue Hua[1,4], Haixiang Sun[3,4*], Bing Yao[2,4*], Chaojun Li[1,4*]

Supplementary figures S1-S6 and supplemental tables 1-2.

Figure S1

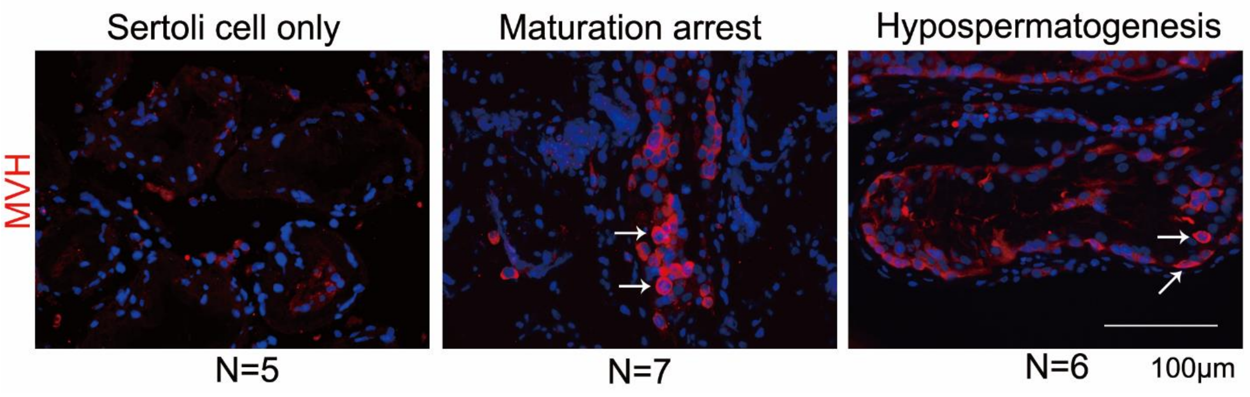

Figure S1. Different types of NOA patients: Sertoli cell only, maturation arrest and hypospermatogenesis. White arrows indicated the positive staining of MVH. The respective number of the three types of NOA patients is 5, 7 and 6. Scale bar: 100μm.

Figure S2

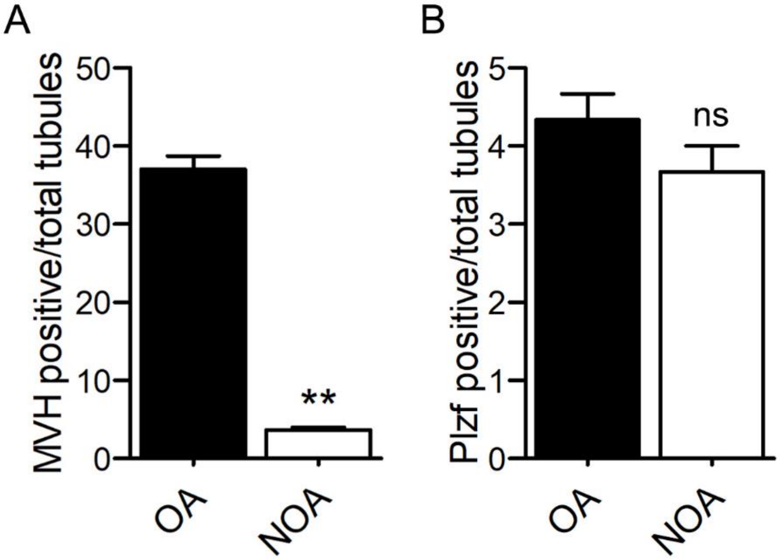

Figure S2. GC number, but not the SSC number, is much lower in NOA patients than that in OA patients. (A and B) Statistical analysis of GC marker MVH-positive staining and SSC marker Plzf-positive staining per tubules of figure 1A and 1B. Data are presented as the mean ± SEM. * p<0.05, **p<0.01, ns: not significant. n=18 of NOA patients and n=5 of OA patients for immunofluorescence respectively.

Figure S3

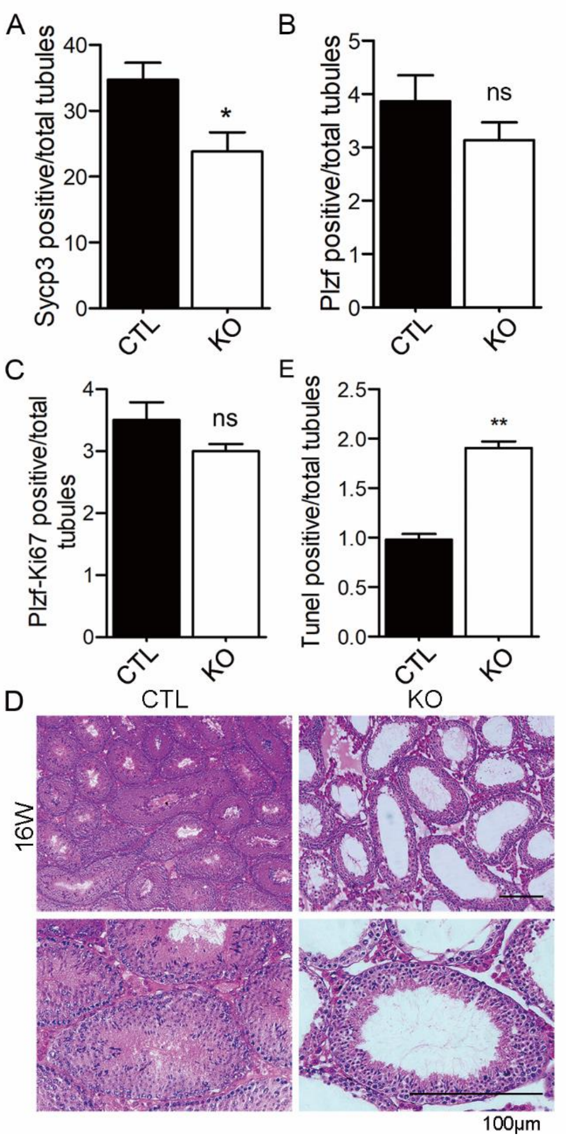

Figure S3. *Ggpps* deletion leads to spermatocyte loss rather than SSCs loss. (A and B) Statistical analysis of positive staining of spermatocyte marker Sycp3 and SSC marker Plzf in CTL and KO mice of figure 3C and 3D. (C) Statistical analysis of Plzf and Ki67 co-staining in CTL and KO mice of figure 3D. (D) H&E staining of mice testis in CTL and KO mice at 16W-old. (E) Statistical analysis of apoptotic cell number by using Tunel assay in CTL and KO mice of figure 3E. Data are presented as the mean ± SEM. * $p<0.05$, ** $p<0.01$, ns: not significant. $n\geq3$.

Figure S4

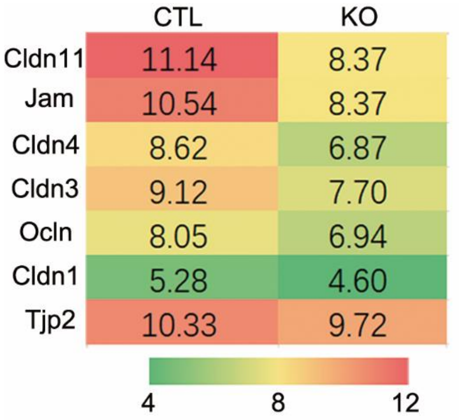

Figure S4. The tight junction-associated protein levels are decreased after *Ggpps* deletion. The analysis of the gene microarray of the primary SC.

Figure S5

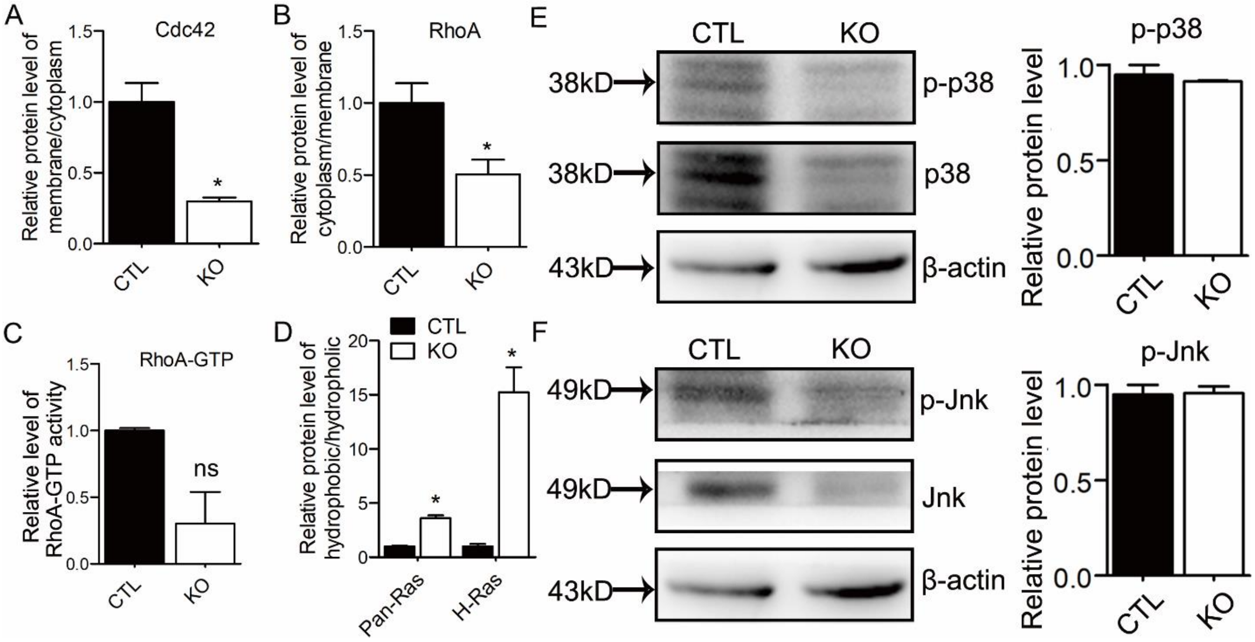

Figure S5. *Ggpps* affects the BTB and cell adhesion via the regulation of protein isoprenylation of the small G-protein. (A and B) Statistical analysis of hydrophobic/hydrophilic protein ratio of Rho family members Cdc42 and RhoA in CTL and KO mice testis of figure 4A and 4B. (C) Statistical analysis of RhoA-GTP activity assay in CTL and KO mice testis of figure 4C. (D) Statistical analysis of hydrophobic/hydrophilic protein ratio of Pan-Ras and H-Ras in primary SC of figure 4D. (E and F) Immunoblot and statistical of p-p38, p38, Jnk and p-Jnk in CTL and KO mice testis. Data are presented as the mean ± SEM. * $p<0.05$, ** $p<0.01$, ns: not significant. $n \geq 3$.

Figure S6

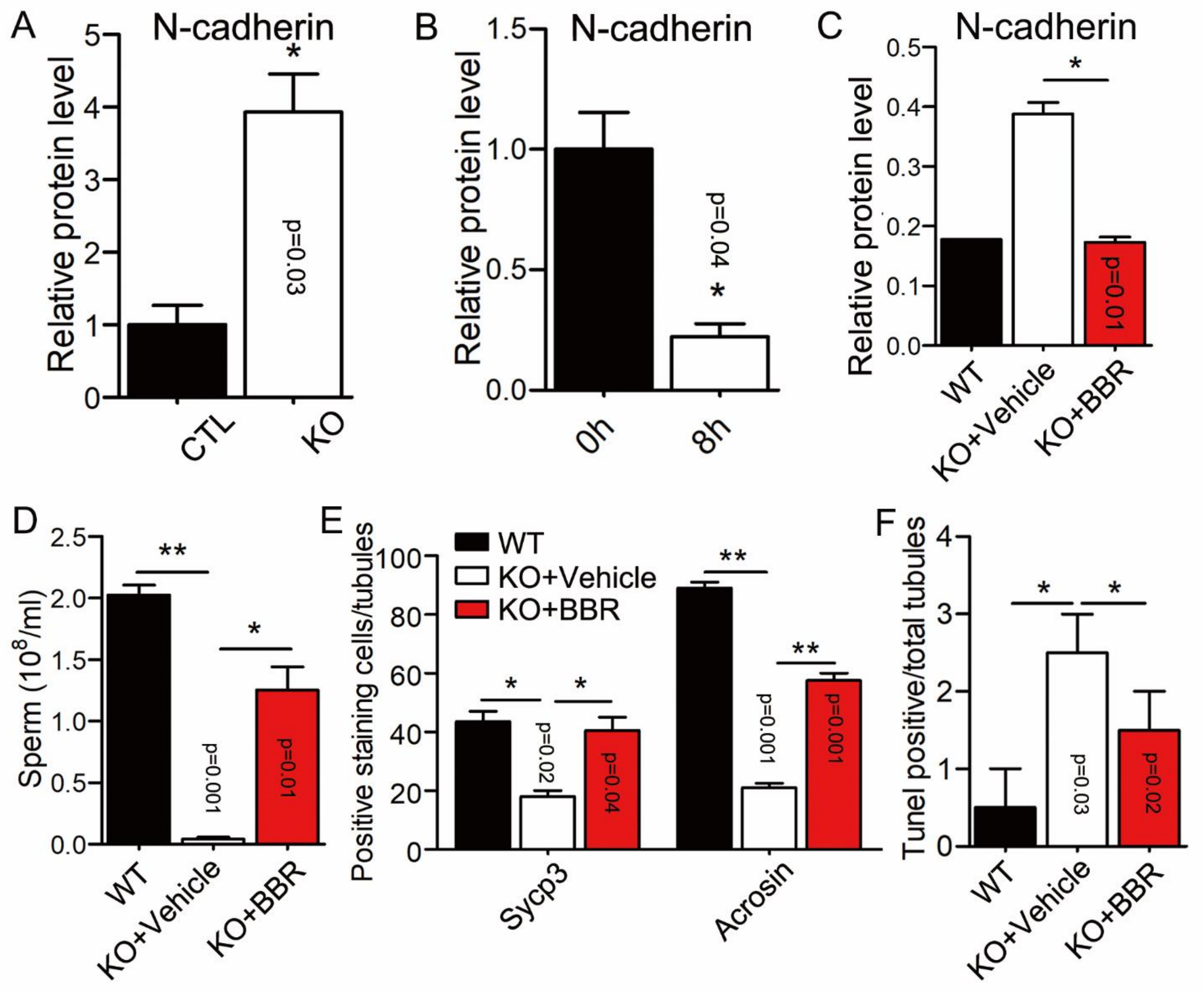

Figure S6. Berberine could partially recover spermatogenesis. (A) Statistical analysis of N-cadherin in CTL and KO mice testis of figure 5A. (B) Statistical analysis of N-cadherin of primary SC in KO mice after berberine treatment of figure 5B. (C) Statistical analysis of N-cadherin in WT and KO mice testis after berberine treatments of figure 5C. (D) Sperm production of epididymis after berberine treatment. (E) Statistical analysis of positive staining of Sycp3 and Acrosin in WT and KO mice after berberine treatment of figure 6E and 6F. (F) Statistical analysis of positive staining of Tunel assay in WT and KO mice after berberine treatment of figure 6G. Data are presented as the mean ±SEM. * $p<0.05$, ** $p<0.01$. $n\geq3$.

Supplemental table S1

The detailed information of the antibodies we used in this work.

Antibody	Source	Catalog number	concentration
MVH	Abcam	ab13840	1 mg/ml
Sycp3	Abcam	ab97672	1 mg/ml
ZO-1	Santa Cruz	sc-10804	200μg/ml
Plzf	Santa Cruz	sc-28319	200μg/ml
Ki67	Abcam	ab15580	1 mg/ml
N-cadherin	Proteintech	22018-1-AP	353μg/ml
GGPPS	Santa Cruz	sc-271679	200μg/ml
GGPPS	Proteintech	14944-1-AP	233μg/ml
E-cadherin	CST	#3195p	1mg/ml
GAPDH	Kangchen Bio-tech	KC-5G4	1mg/ml
Cdc42	Abcam	ab64533	1mg/ml
RhoA	Santa Cruz	sc-418	200μg/ml
Pan-Ras	Santa Cruz	sc-166691	200μg/ml
H-Ras	Santa Cruz	sc-68743	200μg/ml
K-Ras	Santa Cruz	sc-521	200μg/ml
p-ERK	CST	#9106	1mg/ml
ERK	CST	#4695	1mg/ml
α-Tubulin	Boster	BM1452	100ug/vial
β-actin	ABclonal	AC026	1mg/ml
Goat anti-Rabbit IgG Alexa Fluor 594	*Thermo* Scientific™	A11012	2mg/ml
Goat anti-Mouse IgG Alexa Fluor 594	*Thermo* Scientific™	A11032	2mg/ml
Goat anti-Rabbit IgG	*Thermo* Scientific™	A21206	2mg/ml

Alexa Fluor 488			
Goat anti-Mouse IgG Alexa Fluor 488	*Thermo* Scientific™	A21202	2mg/ml

Supplemental table S2

The detailed information of the NOA and OA patients in this work.

NOA	Age	Semen volume (ml)	Sperm number	Mobility (%)	pH
1	27	4.10	0×10^6	0	7.4
2	34	3.20	0×10^6	0	7.4
3	26	4.40	0×10^6	0	7.4
4	36	3.30	0×10^6	0	7.4
5	33	3.60	0×10^6	0	7.4
6	28	3.70	0×10^6	0	7.4
7	30	3.40	0×10^6	0	7.4
8	25	4.00	0×10^6	0	7.4
9	27	3.90	0×10^6	0	7.4
10	32	3.60	0×10^6	0	7.4
11	31	4.20	0×10^6	0	7.4
12	30	4.00	0×10^6	0	7.4
13	38	3.70	0×10^6	0	7.4
14	36	3.20	0×10^6	0	7.4
15	35	3.50	0×10^6	0	7.4
16	26	4.20	0×10^6	0	7.4
17	24	4.50	0×10^6	0	7.4
18	29	4.30	0×10^6	0	7.4

OA	Age	Semen volume（ml）	Sperm number	Mobile number(%)	pH
1	30	4.30	0×10^6	0	7.4
2	32	4.10	0×10^6	0	7.4
3	27	4.60	0×10^6	0	7.4
4	25	4.50	0×10^6	0	7.4

5	35	3.90	0×10^{6}	0	7.4
